# Role of STAT3 in Cancer Metastasis and Translational Advances

**DOI:** 10.1155/2013/421821

**Published:** 2013-10-02

**Authors:** Mohammad Zahid Kamran, Prachi Patil, Rajiv P. Gude

**Affiliations:** Gude lab, Advanced Centre for Treatment, Research & Education in Cancer (ACTREC), Tata Memorial Centre, Kharghar, Navi Mumbai 410210, India

## Abstract

Signal transducer and activator of transcription 3 (STAT3) is a latent cytoplasmic transcription factor, originally discovered as a transducer of signal from cell surface receptors to the nucleus. It is activated by tyrosine phosphorylation at position 705 leading to its dimerization, nuclear translocation, DNA binding, and activation of gene transcription. Under normal physiological conditions, STAT3 activation is tightly regulated. However, compelling evidence suggests that STAT3 is constitutively activated in many cancers and plays a pivotal role in tumor growth and metastasis. It regulates cellular proliferation, invasion, migration, and angiogenesis that are critical for cancer metastasis. In this paper, we first describe the mechanism of STAT3 regulation followed by how STAT3 is involved in cancer metastasis, then we summarize the various small molecule inhibitors that inhibit STAT3 signaling.

## 1. Introduction

**Figure 1:**
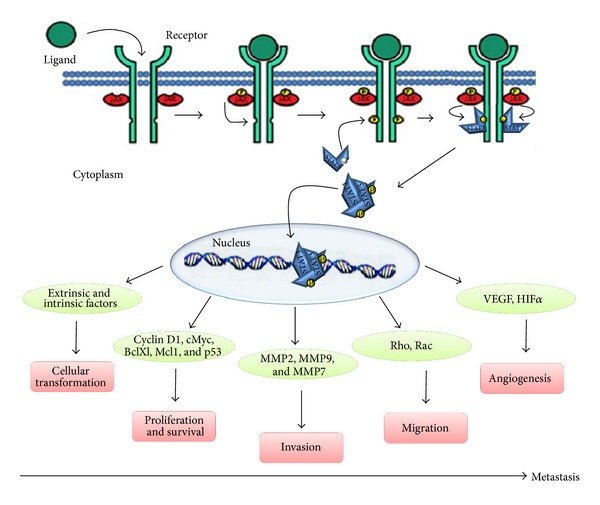
Binding of various ligands to their cognate cell surface receptors, results in phosphorylation of STAT3 molecules that further dimerizes with each other at SH2 domain and gets translocated to the nucleus. Following translocation, the dimerized STAT3 molecule binds to the promoter of target genes and activates their transcription. STAT3 regulate Cyclin D1, cMyc, BclXL, Mcl1 and p53, thereby regulating cellular proliferation and survival. STAT3 directly binds to the promoter of MMP2 and upregulates its expression. Additionally, STAT3 also regulate activity of MMP9 and MMP7. STAT3 regulates cellular migration by modulating the activity of Rho and Rac. Angiogenesis required for tumor growth and metastasis. STAT3 is seen to be regulating angiogenesis by upregulating the activity of VEGF and HIF*α*.

STAT family proteins are latent cytoplasmic transcription factors initially discovered as acute phase response factors in 1994 [1]. These belong to a highly conserved family of protein, and comprise seven members, STAT1 to STAT4, STAT5a, STAT5b, and STAT6 [2, 3]. In resting cells, STATs are generally located in the cytoplasm in their inactive state. Phosphorylation of specific tyrosine residue is an essential step for STAT activation. Once activated, STAT dimerizes to other STATs by reciprocal SH2 phosphotyrosine interaction, leading to its translocation into the nucleus followed by its binding to the specific enhancer elements for initiation of transcription [2, 3] (Figure 1). Studies from knockout mice revealed that individual STAT protein is essential for various normal physiological functions such as embryonic development, cell differentiation, immune response, and organogenesis [4] (Table 1).

All seven human STAT proteins range between 750 and 850 amino acids and are located on three chromosomal clusters [3, 5, 6]. The genes encoding STAT1 and STAT4 are located on chromosome 2, STAT2 and STAT6 on chromosome 17, and STAT3, STAT5a, and STAT5b on chromosome 12. Structural studies revealed that all STAT family proteins have seven structurally and functionally conserved domains including the amino-terminal domain (NH2), coiled-coil domain, DNA-binding domain (DBD), linker domain (Lk), SH2 domain, tyrosine activation domain (Y), and transactivation domain (TAD) that have distinct roles [3]. The amino-terminal domain spans up to 125 amino acid residues and is involved in the formation of homotypic dimers among unphosphorylated STATs in resting cells. The coiled-coil domain interacts with various regulatory proteins and other transcription factors. The DNA-binding domain is involved in the direct binding of STATs to the corresponding sites in gene promoter. The linker domain adjacent to DNA binding maintains an appropriate conformation between the dimerization domain and DNA-binding domain. Next, the SH2 domain, which is a highly conserved domain, is involved in formation of active STAT dimer. The tyrosine activation domain consists of a conserved tyrosine residue usually nearby position 700. Phosphorylation of this tyrosine residue is essential for STAT activation. Finally, the carboxyl-terminal transactivation domain, a highly variable domain in context of length and sequence among STAT family members, regulates transcriptional activation of target genes through interaction with other transcriptional regulators. Conserved serine phosphorylation site is present in many transactivation domains. Phosphorylation of this serine residue is required for maximal transcriptional activity [7, 8].

Among seven mammalian STAT proteins, persistent activation of STAT3 followed by STAT5 is frequently detected in majority of human cancer cell lines and tumor tissues [9, 10]. These include breast cancer, lung cancer, pancreatic cancer, head and neck cancer, prostate cancer, ovarian cancer, melanoma, leukaemias, and lymphomas. It is observed that aberrant STAT3 activation in tumor cells is associated with cell proliferation, cell survival, invasion, angiogenesis, and metastasis [11] (Figure 1). Conversely, targeting STAT3 activation inhibits tumor growth and metastasis both *in vitro* and *in vivo* without affecting normal cells, thus suggesting that STAT3 could be a valid molecular target for cancer therapy [12].

## 2. Mechanisms of STAT3 Activation

STAT3 is activated by phosphorylation of a single tyrosine residue located at position 705. Various tyrosine kinases that catalyze this phosphorylation include such receptors with intrinsic tyrosine kinase activity as epidermal growth factor (EGFR), vascular endothelial growth factor receptor (VEGFR), platelet derived growth factor receptor (PDGFR), and colony stimulating factor-1 [13, 14]. Along with the nonreceptor tyrosine kinases such as Src and abl, cytokine receptors such as IL6R that show association with JAKs also catalyse the tyrosine phosphorylation [1, 15, 16]. Apart from tyrosine kinases, various serine kinases such as MAPK (p38MAPK, ERK, JNK), PKC^*δ*^, mTOR, and NLK have been reported to phosphorylate STAT3 at serine position 727 which is required for STAT3 maximal transcriptional activity [8, 17–20]. Additionally, STAT3 is also acetylated on a single lysine residue located at position 685 by histone acetyl transferase p300 [21]. This acetylation appears to regulate both transcriptional activity and homodimer stability [21]. Other factors such as UV radiation or sun light, carcinogen, stress, smoke, and infection are also known to play a significant role in STAT3 activation.

## 3. Regulation of STAT3 Activity

STAT3 activation is negatively regulated through numerous mechanisms which involves the following.

### 3.1. Tyrosine Phosphatases

Since tyrosine kinases play an important role in STAT3 activation, it is not surprising that tyrosine phosphatases are likely to play a role in STAT3 deactivation. These include classical protein tyrosine phosphatases (PTPs), dual-specificity phosphatases, and low molecular weight phosphatases [22]. Although these protein tyrosine phosphatases show little sequence similarity, these proteins exhibit similar tertiary structure and are characterized by presence of signature motif VHCSXGXGR[T/S]G [23, 24]. The classical PTPs are divided into 2 groups, the transmembrane tyrosine phosphatase CD45 and nontransmembrane PTPs, including SH2-domain-containing SHP1 and SHP2, phospho-tyrosine phosphatase 1B (PTP1B), and T cell-protein tyrosine phosphatase (TC-PTP). Increased JAK2 and STAT3 phosphorylation in response to targeted disruption of the CD45 gene was reported by Irie-Sasaki and coworkers [25]. Also, loss of SHP1 enhances JAK3 and STAT3 phosphorylation [26] while hepatocytedeletion of SHP2 promotes STAT3 signaling [27].

Apart from PTPs, dual-specificity phosphatases dephosphorylate both phosphotyrosine and phosphoserine/phosphothreonine. STAT3 is phosphorylated in response to angiotensin II at tyrosine and serine residues in vascular smooth muscle cells [28]. PP2B, a dual-specificity phosphatase, dephosphorylates STAT3 tyrosine phosphorylation, while protein phosphatase 2A (PP2A) dephosphorylates STAT3 serine phosphorylation [28]. 

Higher expression of low molecular weight phosphatase is generally observed in megakaryoblastic cells. In DAM1 megakaryocytic cells, low molecular weight phosphatase dephosphorylates STAT5 by interacting with its C-terminal domain [29].

### 3.2. Protein Inhibitors of Activated STATs (PIAS)

The PIAS family proteins consisting of five members, namely, PIAS1, PIAS3, PIASy, PIASxa, and PIASxb, have been proposed to regulate the activity of many transcription factors, including STATs [30–35]. Amino-terminal region of all PIAS proteins is characterized by conserved LXXLL signature motif [31]. Other conserved elements of PIAS proteins include zinc binding domain, acidic domain, and serine/threonine rich regions; however, PIASy lacks serine/threonine rich domain [31]. PIAS mediated gene regulation involves direct blocking of DNA-binding activity of transcription factors, recruiting transcriptional corepressors or coactivators, and promoting protein sumoylation [30]. It has been proposed that PIAS1, PIAS3 and PIASx interact with STAT1, STAT3, and STAT4 respectively [32, 33, 35]. PIASy also interacts with STAT1 [34]. PIAS1 and PIAS3 repress transcriptional activity of STAT1 and STAT3, respectively [32, 33], whereas PIASx and PIASy repress the transcriptional activity of STAT1 and STAT4 [34, 35].

### 3.3. Suppressors of Cytokine Signaling (SOCS) Proteins

SOCS family proteins are inducible, with SH2 domain containing inhibitors of cytokine signaling, and consist of eight members: CIS along with SOCS1 to SOCS7 [36–38]. These inhibit STATs signaling in three ways, either by binding their SH2 domain to JAKs (in case of SOCS1), by binding to receptor cytoplasmic domain (in case of SOCS3), or by competing with STAT-SH2 domains for the recruitment to the receptor complex (CIS, SOCS2) [39]. In addition, SOCS proteins also induce proteasomal degradation pathway through SOCS box [40]. 

## 4. Role of STAT3 in Cancer Metastasis

Cancer metastasis is a complex, multistep process in which tumor cells primarily invades surrounding tissue and basement membrane, thus entering into blood circulation; while surviving during circulation, the tumor cells extravasate and adhere into distant organ and induce angiogenesis to form secondary tumor. Compelling evidence supports the fact that STAT3 activation plays a critical role in every step of metastasis including cell proliferation and survival, invasion, migration, and angiogenesis (Figure 1). The detailed explanation of how STAT3 regulates different steps of metastasis is described below.

### 4.1. STAT3 and Cell Transformation

Malignant transformation of cells by various protein tyrosine kinases, oncogenes, and viruses is mediated through STAT3 activation. Bromberg and coworkers for the first time reported that STAT3 could itself be responsible for cellular transformation [41]. By using NIH3T3 cell, Turkson and coworkers showed that STAT3 activation by Src may contribute to cell transformation by preventing apoptosis, thereby indirectly increasing cell numbers [42]. Yu and coworkers demonstrated that STAT3 is activated in interleukin-6 induced transformation in mouse skin epithelial cells. [43]. Miranda and coworkers unveiled the role of STAT3 in *in vitro* transformation that was triggered by TRK oncogene [44]. Similarly, the transformation of NIH3T3 fibroblast by RET/PTC tyrosine kinase was mediated through the activation of STAT3 [45]. Hepatitis C virus core protein, large tumor antigen of simian virus 40, and herpesvirus Saimiri STP-A oncoprotein have all shown their respective roles in transforming the cells through activation of STAT3 [46–48]. In contrast, targeting STAT3 decreases malignant transformation susceptibility of a number of cell types [49]. Thus, these observations strengthen the role of STAT3 in malignant transformation.

### 4.2. STAT3 and Cellular Proliferation and Apoptosis

In addition to being involved in cellular transformation, STAT3 also participates in cellular proliferation and survival. Both cMyc and cyclin D1 are required for regulation of G1 phase of cell cycle [50]. Evidence indicates that constitutive STAT3 signalling is associated with upregulation of cyclin D1 and cMyc expression, contributing to accelerated cell-cycle progression. STAT3 has also been shown to upregulate the expression of growth promoting gene pim-1 [51]. Consistent with its role in cellular proliferation, various studies have demonstrated that STAT3 signaling provides survival signals and suppresses the apoptosis in cancerous cells. These effects are mediated through the expression of Bcl2, BclxL, Mcl1, surviving, and cIAP2 [52]. In addition, STAT3 negatively regulates the expression of p53, which is considered to be the most common inhibitor of cellular proliferation as well as inducer of apoptosis [53]. However, recent studies suggest that STAT3 can also act as a proapoptotic factor, especially during postlactation regression where LIF acts as the only activator of STAT3 to cause apoptosis in mammary glands [54]. In addition to pro-apoptotic function of STAT3, some studies suggested that loss of STAT3 promotes cellular proliferation and transformation [55].

### 4.3. STAT3 and Cellular Invasion

Invasion to extracellular matrix is one of the key steps in tumor growth and metastasis formation. Several lines of evidence strongly implicate that STAT3 plays a crucial role in this complex multistep process by regulating the matrix metalloproteinases (MMPs). In cutaneous squamous cell carcinoma, overexpression of phosphorylated STAT3 correlated with increased invasion, and metastasis [56]. In contrast, targeting STAT3 blocks tumor growth, invasion and metastasis formation in variety of cell lines both *in vitro* and *in vivo* [57]. Furthermore, STAT3 knockdown using ShRNA reduced pancreatic cancer cell invasiveness and MMP-7 expression in nude mice [58]. Constitutively activated STAT3 protein in melanoma could directly bind to the promoter of MMP-2 gene, thus upregulating its expression [59]. Similarly, STAT3 activation also regulated the expression of matrix metalloproteinases MMP-9 and MMP-1 [60, 61]. All these data show that STAT3 actively promotes the rate of cellular invasion.

### 4.4. STAT3 and Cellular Migration

Cellular migration is a central step for many biological processes, including embryogenesis, cell invasion, and cancer metastasis. Cumulative evidences has indicated the role of STAT3 in cellular migration under normal as well as pathological conditions. Sano and coworkers for the first time reported that STAT3 plays a crucial role in wound healing and cellular migration in cultured keratinocytes [62]. In SKOV3 cells, Silver and coworkers demonstrated that STAT3 signaling is required for cell motility *in vitro* and that depletion of STAT3 using siRNA reversed the condition, thus reducing the rate of cellular migration [63]. On the other hand, stathmin, an oncoprotein 18, binds to *α*/*β*-tubulin heterodimers and is involved in microtubule depolymerisation. Ng and coworkers reported that STAT3 interacts with stathmin and modulates cell migration and microtubule dynamics [64]. Similarly, loss of STAT3 expression results in a random mode of migration, while STAT3 modulates Rac1 activity to maintain directional persistence during migration [65]. In another work by Debidda and coworkers, the role of STAT3 is demonstrated in Rho GTPase-regulated cell migration as well as proliferation [66]. Accordingly, persistent STAT3 activation enhances migratory potential of prostate epithelial cells through integrin*β*6 [67].

### 4.5. STAT3 and Tumor Cell Intravasation and Survival during Circulation

Following degradation of basement membrane, tumor cells enter the circulatory or lymphatic system (intravasation), survive during circulation, and extravasate into the new potent organs where they adhere to form detectable metastasis. As metastatic tumor cells enter the blood vessels, they are subjected to various nonspecific forces such as mechanical stress, hemodynamic turbulence, loss of adhesion-induced cell death, and cell mediated cytotoxicity [68]. As a result, a very low percentage of tumor cells survive during circulation which further establishes micrometastasis in distant organs. STAT3 activation has a major role in protecting the tumor cells from body's immune surveillance during their transit through circulation. Nguyen and coworkers observed that activation of STAT3 signaling in tumor cells or in inflammatory immune cells modulates secretion of various inflammatory factors such as IL6 and TNF*α* that act as an immunosuppressors, and increase the probability of survival of tumor cells [69]. Moreover, STAT3 activation in tumor microenvironment also reduces the activity of NK cells, thereby protecting tumor cells during circulation [70]. Additionally, tumor cells make association with platelets which protect them from the stresses of shear flow [71]. Thus, STAT3 activation increases the number of surviving tumor cells that invade distant potent organs to form secondary tumor. 

### 4.6. STAT3 and Angiogenesis

Angiogenesis, the formation of new blood vessels from preexisting vasculature, is an essential step for tumor growth and metastasis. The most potent angiogenic molecule is vascular endothelial growth factor (VEGF) [72, 73]. VEGF secreted from tumor cells binds to transmembrane receptor tyrosine kinases of endothelial cells and participates in neovascularisation. STAT3 is regarded as a direct transcriptional activator of VEGF gene [74]. Niu and coworkers reported that constitutive activation of STAT3 upregulates VEGF expression and tumor angiogenesis in melanoma cells [75]. Additionally, Wei and coworkers also reported that STAT3 activation regulates VEGF expression and angiogenesis in human pancreatic cancer cells [76]. However, in contrast to this, targeting STAT3 blocks both VEGF expression and angiogenesis [77]. Besides this, various reports have also shown that STAT3 activation regulates VEGF receptor signaling in endothelial cells [78, 79]. Inhibiting STAT3 signaling in endothelial cells prohibits their migration and vessel formation [79]. STAT3 has also been reported to induce expression of hypoxia-inducible factor-1*α* (HIF1*α*), another key mediator of angiogenesis [80]. In hypoxic conditions, both STAT3 and HIF1*α* bind simultaneously to the VEGF promoter leading to its maximum transcriptional activation and angiogenesis [81].

### 4.7. STAT3 and Tumor Microenvironment

Tumor cells adapt to and modify their surrounding microenvironment [82]. It is observed that tumor cells having constitutively active STAT3 signalling recruit immune cells and subvert their function for self-benefit [82]. This is achieved by increased production of immunosuppressive agents via immunosuppressive cells such as T-helper cells, dendritic cells, and macrophages and reduction in immune activation signals. STAT3 is observed to be a potent negative regulator of T1 helper cells. Several studies suggested that inhibition of STAT3 activation promotes the release of proinflammatory cytokines, while a mutant having constitutively active STAT3 in fibroblasts suppressed the LPS induced pro-inflammatory response [70, 83–85]. This suggests that STAT3 activation negatively regulates the activity of immune stimulating molecules. Dendritic cells are known to be involved in antitumor activity. However, the dendritic cells surrounding the tumor cells are partially differentiated and thus lack MHC class II molecules [86, 87]. The tumor secreted factors such as IL6, IL10, and VEGF are responsible for this partial differentiation of dendritic cells, hence reducing their antigen presenting ability [70]. Reports suggest that the tumor cells lacking STAT3 activation could efficiently produce the proinflammatory factors that promote the maturation and antigen presenting ability of dendritic cells. Apart from dendritic cells, other immune effector cells such as macrophages, neutrophils, natural killer cells, and regulatory T cells, in response to the growth factors released by tumor cells, show constitutive activation of STAT3, thus limiting their own ability of immune surveillance [88]. Additionally, stromal cells, in response to surrounding tumor cell secretions, upregulate their SDF-1/CXCL12 receptors which results in infiltration of endothelial progenitor cells that enhance metastatic spread of tumor cells [82]. Thus, all these enlighten the fact that STAT3 mediates a bidirectional communication with immune cells. The use of STAT inhibitors has been shown to reduce the immunosuppressive response, thus upregulating the anti-tumor ability of immune effector cells. Although STAT3 is considered as a primary target to overcome immunosuppression, more recently other signal transduction pathways such as MAPKs have also attracted attention in order to prevent immunosuppression. 

## 5. STAT3 Inhibitors: Translational Advances

STAT3 is excessively active in many cancers and plays a central role in tumorigenesis. Several lines of evidence have implicated that inhibition of STAT3 with a dominant negative form of STAT3 or other inhibitors attenuates the proliferation and survival of a wide variety of cancers with little or no effects on normal cells [12]. While inactivation of STAT3 leads to embryonic lethality in mice, its function is dispensable in many adult tissues. Collectively, these lines of evidence have validated STAT3 as a target for cancer therapy. Several molecules have been identified that can block STAT3 activation in a variety of preclinical models both *in vitro* and *in vivo* (Table 2). These include the following.

### 5.1. Curcumin

Curcumin, an active component of turmeric, has been demonstrated to have anticancerous, anti-inflammatory, and antioxidant properties. Curcumin inhibits STAT3 activation in various cell lines including multiple myeloma [89], pancreatic cancer cell lines [90], Hodgkin's lymphoma [91], head and neck squamous cell carcinoma [92], primary effusion lymphoma [93], human chronic myelogenous leukaemia [94] and ovarian cancer [95]. Further, a small molecule curcumin analog, FLLL32, induces apoptosis in melanoma cells via STAT3 inhibition without altering STAT1 signaling [96].

### 5.2. Resveratrol

Resveratrol (3,5,4′-trihydroxystilbene), a naturally occurring polyphenolic phytoalexin has anti-inflammatory and anti-oxidant properties. Jang and coworkers reported that resveratrol treatment inhibited the development of preneoplastic lesions in carcinogen-treated mouse mammary glands in culture and tumorigenesis in a mouse skin cancer model [97]. Further mechanistic studies revealed that resveratrol inhibited tumor growth and induced apoptosis by suppressing STAT3 signaling. Additionally, Kotha and coworkers reported that resveratrol inhibited Src and STAT3 signaling and induced apoptosis of malignant cells (NIH3T3/v-Src fibroblasts, human breast cancer MDA-MB-468 and MDA-MB-231, prostate cancer DU145, or human pancreatic cancer Panc-1 and Colo-357) containing activated STAT3 protein [98]. Other reports show that resveratrol inhibits STAT3 activation in many other cancer cell lines including multiple myeloma [99], endothelial cells [100], medulloblastoma [101], and glioblastoma [102].

### 5.3. Flavopiridol

Flavopiridol, a flavonoid derived from an Indian plant, is a potent cyclin-dependent kinase inhibitor [103]. At higher concentrations, it inhibits the activity of receptor tyrosine kinases (EGFR), receptor associates tyrosine kinases (pp60 Src), and signal transducing kinases (PKC and Erk-1) [103]. Flavopiridol induces apoptosis in many human cancer cell lines *in vitro* and has potent antitumor activity *in vivo* against human leukemia and lymphoma xenografts [104, 105]. Studies by Lee and coworkers demonstrated that flavopiridol disrupts STAT3/DNA interaction, attenuates STAT3 directed transcription, and downregulates Mcl1, an antiapoptotic protein present downstream of STAT3 [104]. Flavopiridol in combination with bortezomib also inhibits activity of STAT3 and STAT5 and induces apoptosis in chronic myeloid leukemia [106].

### 5.4. Cucurbitacin Derivatives

Cucurbitacin, and their derivatives are triterpenoid compounds isolated from various plant families, such as Cruciferae or Cucurbitaceae. They exhibit diverse biological activities including anti-inflammatory and anticancer effects. Several cucurbitacin derivatives have been reported to show their effect on STAT3 signaling. JSI-124, previously identified as cucurbitacin I, reduced the levels of phosphotyrosine of constitutively activated STAT3 in many human cancer cell lines including lung and breast carcinomas [107, 108]. Cucurbitacin B, another derivative suppresses tumor growth in pancreatic cancer cells by inhibiting JAK/STAT pathway and synergistically increases antiproliferative effects of gemcitabine *in vitro* [109]. Cucurbitacin Q also inhibits STAT3 activation and induces apoptosis without inhibiting JAK2, Src, Akt, Erk, or JNK activation in human and murine tumors that contain constitutively activated STAT3 (i.e., A549, MDA-MB-435, and v-Src/NIH 3T3) [110]. Cucurbitacin E inhibits STAT3/p53/p21 signaling and induces apoptosis via Fas/CD95 and mitochondria-dependent pathways in human bladder cancer T24 cells [111].

### 5.5. Deoxytetrangomycin

Deoxytetrangomycin or STA1 was discovered by structure-based virtual database screening of the National Cancer Institute (NCI) database. It inhibits STAT3 dimerization, STAT3 nuclear translocation, STAT3 DNA binding, and STAT3-dependent luciferase activity in MDA-MB-435s breast cancer cells [112]. 

### 5.6. Cyclopentenone Derivatives

Weidler and coworkers identified CPDHC(1), a novel cyclopentenone derivative as an inhibitor of IL6 dependent JAK/STAT pathway while screening fungal extracts with a cell based reporter assay [113]. CPDHC(1) inhibits STAT3 and STAT1 tyrosine phosphorylation and STAT3 serine phosphorylation in HepG2 cells [113].

### 5.7. N-Acyl Homoserine Lactone

N-Acyl homoserine lactones are a class of signaling molecules involved in bacterial quorum sensing [114]. The quorum-sensing signal molecule of *P. aeruginosa*, N-(3-oxododecanoyl)-L-homoserine lactone (OdDHL), has been reported to modulate inflammation and immune responses in mammals. In addition, Li and coworkers reported that OdDHL blocks proliferation and induces apoptosis in human breast cancer cell lines such as BR293, MCF-7, and MDA-MB-468 [115]. Further studies on the mode of action of OdHL revealed that it has no significant effect on MAPK cascades, partially inhibits the Akt/PKB pathway, and suppresses STAT3 activity [115]. 

### 5.8. Indirubin Derivatives

Indirubin, an active constituent of a Chinese herbal medicine, is used for the treatment of chronic myelogenous leukemia and has been shown to be a potent inhibitor of cyclin dependent kinases [116]. Reports suggest that indirubin or its derivatives inhibit cellular proliferation and induce apoptosis in many human cancers [117]. Nam and coworkers reported that indirubin derivative E804 suppressed STAT3 tyrosine phosphorylation by inhibiting upstream cSrc kinase activity [118]. The antiapoptotic proteins Mcl-1 and survivin were down-regulated upon E804 treatment followed by induction of apoptosis in human breast cancer cells [118]. Apart from E804, 6-bromoindirubin-3-oxime, a bromo derivative of indirubin selectively inhibits JAK/STAT3 signaling in human melanoma cells [119]. Similarly, indirubin-3'-monoxime, a chemical derivative of indirubin, was found to inhibit phosphorylation of STAT3 and STAT1 by blocking the activity of the FGFR1 tyrosine kinase in myeloid leukemia cell line KG-1a [120].

### 5.9. Tyrphostins

Tyrphostins represent a novel class of selective protein tyrosine kinase inhibitors that have antitumor activity both *in vitro* and *in vivo*. Ni and coworkers reported that Janus kinase inhibitor, tyrphostin AG490, inhibited the constitutive activation of STAT3 and suppressed the growth of human prostate cancer cells [121]. AG490 also inhibits STAT3 signaling in multiple myeloma [122] and Hodgkins lymphoma [123]. Similarly, Nielsen and coworkers showed that AG490 blocked the constitutive activation of STAT3 and inhibited both spontaneous and interleukin 2-induced growth of mycosis fungoides tumor cells [124]. Another analogue of tyrphostin, AG17, inhibited STAT3 phosphorylation and induced apoptosis in classical Hodgkin lymphoma cells [125].

### 5.10. JAK Inhibitors

JAK/STAT3 signaling is deregulated in majority of human cancers and promotes tumor growth and metastasis. While direct inhibition of STAT3 by small molecule inhibitors is difficult, targeting JAK kinases by using their inhibitors is one of the approaches to inhibit STAT3 signaling. JAK family of kinases comprises four members JAK1, JAK2, JAK3, and TYK2. Various JAK inhibitors have been tested in clinical trials for patients with myelofibrosis and other diseases such as cancer (Table 3). Ruxolitinib, an oral JAK1 and JAK2 inhibitor, is the first JAK inhibitor that has been approved by the FDA for the treatment of myelofibrosis [126]. Currently, ruxolitinib is also in phase II trials for the treatment of haematological malignancies and prostate cancer [127]. Another JAK inhibitor, SAR302503, is currently in phase I clinical trial for the treatment of solid tumors. Pacritinib, a JAK2 inhibitor is in a phase III trial to treat myelofibrosis and in several phase I/II trials in other hematologic malignancies [128]. Another JAK2 inhibitor AZD1480 potently blocks STAT3 signaling and, currently in phase I trial in treatment of solid tumors [129]. Other JAK inhibitors such as BMS-911543, AC-430, and CEP-33779 are either in preclinical stages or in early stages of development (Table 4).

### 5.11. Platinum-Containing Compounds

Platinum-containing compounds such as cisplatin, carboplatin, and oxaliplatin are among the most widely used chemotherapeutic drugs. The mechanism of action of these drugs involves cross linking to purine DNA bases, which leads to apoptosis [130]. It regulates several signaling pathways such as MAPK and PI3K/Akt [131]. Song and coworkers reported that cisplatin inhibits JAK2/STAT3 signaling in cancer cells [132]. Other novel platinum containing complexes were also identified and reported as STAT3 inhibitors. The novel platinum-containing compounds such as CPA-1 and CPA-7 inhibit STAT3 signaling and suppress its biological functions in malignant cells that harbour constitutive STAT3 expression [133]. Another platinum (IV) compound, IS3 295, selected from the NCI diversity set appears to have STAT3 inhibitor activity [134]. Results suggested that IS3 295 selectively inhibits STAT3 signaling pathway, thereby inducing cell cycle arrest at G0/G1 phase and apoptosis [134].

### 5.12. Atiprimod

Atiprimod is a small molecule that belongs to the azaspirane class of drugs. They are cationic amphiphilic agents that exhibit anti-inflamatory, antiproliferative, and antiangiogenic properties. Choudhari and coworkers have shown that atiprimod inhibits protein kinase B (Akt) and STAT3 signaling, thereby inhibiting proliferation and inducing apoptosis in hepatocellular carcinoma [135]. Atiprimod was also found to be involved in inhibiting STAT3 phosphorylation and inducing apoptosis in multiple myeloma cells U266-B1 [136]. In mantle cell lymphoma (MCL), atiprimod was responsible for induction of apoptosis via mitochondrial pathway [137]. A mechanistic study revealed that atiprimod treatment activates JNK signaling and inhibits NF-KB and STAT3 activities in MCL cells [137]. Similarly, in acute myeloid leukemia, atiprimod inhibits proliferation and induces apoptosis by suppressing STAT3 and STAT5 phosphorylation [138].

### 5.13. Pentoxifylline

Pentoxifylline (PTX), a methyl xanthine derivative, is a FDA approved drug for treatment of peripheral vascular disease [139]. It is a nonspecific phosphodiesterase inhibitor that elevates cAMP in the cells and exhibits potent antimetastatic and antiangiogenic activities against many human cancers *in vitro* as well as *in vivo* [140]. PTX at subtoxic doses inhibits proliferation, migration, and invasion of A375 human melanoma cells *in vitro* and tumor growth *in vivo* [140]. Further mechanistic study revealed that PTX inhibits STAT3 phosphorylation and DNA binding in melanoma cells and that this inhibition was partly mediated through the inhibition of upstream kinases pJAK1 and pJak2 [141]. PTX has also been shown to inhibit STAT3 phosphorylation in B16F10 melanoma and A549 lung carcinoma (our unpublished data).

### 5.14. STAT3 Decoy Oligonucleotide

STAT3 decoy comprises a 15 bp double-stranded oligonucleotide, which corresponds closely to the STAT3 response element in the c-fos promoter and binds competitively to STAT3 [142]. STAT3 decoy inhibited proliferation and STAT3 mediated gene expression in squamous cell carcinoma of the head and neck *in vitro* as well as in a xenograft model *in vivo* [142, 143]. Sen and coworkers conducted first phase 0 clinical trial using STAT3 decoy in head and neck cancer and reported that intratumoral administration of STAT3 decoy oligonucleotide abrogates target gene expression in patients with head and neck squamous cell carcinoma [144]. STAT3 decoy has also been shown to inhibit the growth of human lung cancer and glioma [145, 146]. 

## 6. Conclusion and Future Perspectives

Although in normal cells STAT3 expression is very transient and tightly regulated, its persistent activation has been confirmed clinically in majority of tumor samples. This persistent STAT3 activation plays a central role in tumorigenesis and provides favourable conditions to the tumor cells to undergo metastasis while being involved in cellular proliferation, invasion, migration, and angiogenesis. In contrast to this, blocking STAT3 signaling in tumor cells inhibits tumor growth, angiogenesis, and metastasis without affecting normal cells, thus confirming STAT3 as a potential target for cancer therapy. Despite the different approaches to identify small molecules that effectively inhibit STAT3 signaling, further studies will be needed to make these molecules more effective for improved clinical outcomes. We believe that this is possible by using interdisciplinary approaches of rational drug design and/or high throughput screening or by optimization of various combination therapies. 

## Figures and Tables

**Table 1:** Phenotype of STATs knockout mice.

Targeted gene	Phenotype
STAT1	Compromised innate response to microbial pathogens and viruses.
STAT2	Increased susceptibility to viral infection and a loss of biological response to type I IFN.
STAT3	Early embryonic lethality.
STAT4	Impaired natural killer cell cytotoxicity and Th1 cell response.
STAT5a and 5b	No mammary gland development or lactogenesis.
STAT6	No Th2 cells development.

**Table 2:** Small molecule STAT3 inhibitors and their structure.

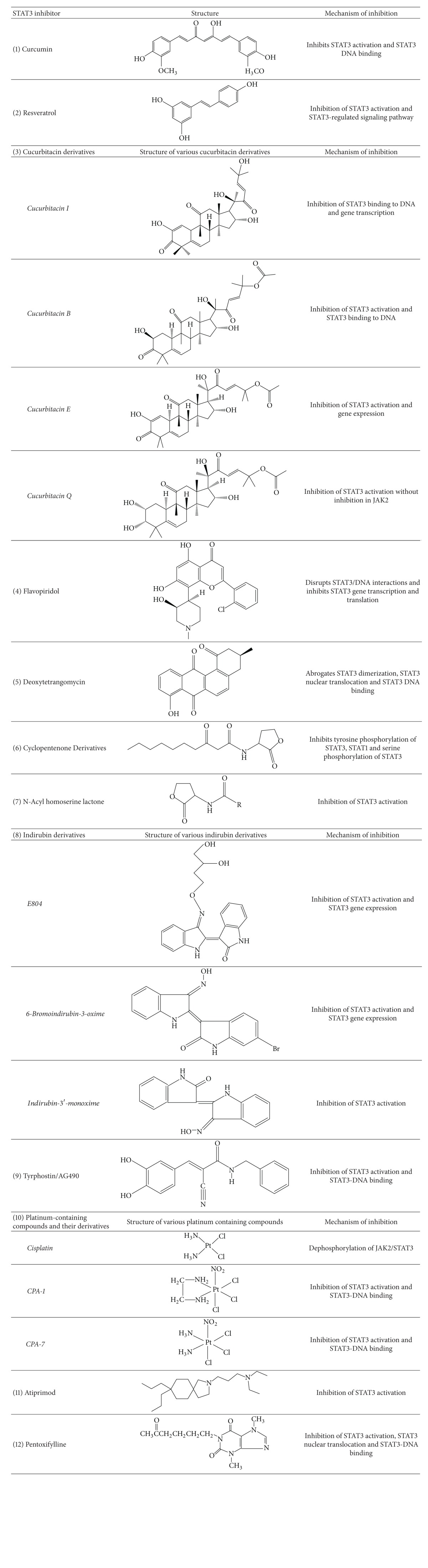

**Table 3:** Selected JAK inhibitors currently being evaluated in clinical trials for autoimmunity and cancer.

Inhibitor	Targeted JAK	Phase in development
Ruxolitinib	JAK2, JAK1	FDA approved for myelofibrosis Phase II in prostate cancer, hematological malignances
Tofacitinib	JAK3, JAK1	FDA approved for rheumatoid arthritis
SAR302503	JAK2, JAK1	Phase I in solid tumors Phase III in myelofibrosis
CYT387	JAK2, JAK1	Phase II in myelofibrosis
Pacritinib	JAK2	Phase II in hematologic malignancies
AZD1480	JAK2, JAK1	Phase I in solid tumors
INCB028050	JAK2, JAK1	Phase II in rheumatoid arthritis

**Table 4:** Selected JAK inhibitors in preclinical stage.

Inhibitor	Targeted JAK	Indication
BMS-911543	JAK2	Myelofibrosis
AC-430	JAK2	Rheumatoid arthritis, lymphoma
CEP-33779	JAK2	Rheumatoid arthritis
R723	JAK2	Myeloproliferative neoplasias
